# Endoscopic ultrasound-guided hepaticogastrostomy with antegrade stenting using a catheter-like delivery system: a novel technique completely omitting the fistula dilation step

**DOI:** 10.1055/a-2587-9552

**Published:** 2025-05-14

**Authors:** Tadahisa Inoue, Rena Kitano, Tomoya Kitada, Shun Futagami, Masato Yano, Jun Arai, Kiyoaki Ito

**Affiliations:** 1 12703 Department of Gastroenterology, Aichi Medical University, Aichi, Japan

When the transpapillary approach is not feasible, endoscopic ultrasound-guided hepaticogastrostomy (EUS-HGS) is a valuable option for malignant distal biliary obstruction (MDBO) 1 . Recent studies suggest that adding antegrade stenting (AS) to HGS (EUS-HGAS) may provide longer stent patency than HGS alone 2 3 . However, regardless of AS addition, a significant challenge in EUS-guided transgastric drainage is the fistula dilation step. Fistula dilation is technically demanding and time-consuming, in addition the risk of bile leakage between dilation and stent placement is a major concern.

To address this issue, we propose a novel EUS-HGAS technique that completely omits the fistula dilation step using a catheter-like delivery system for metal stents. This novel delivery system allows contrast injection for cholangiography while maintaining sufficient flow, has a diameter equivalent to small-caliber catheters (5.7-Fr), and features a tapered tip for smooth insertion, similar to dilation catheters ( Fig. 1 ).

**Fig. 1:**
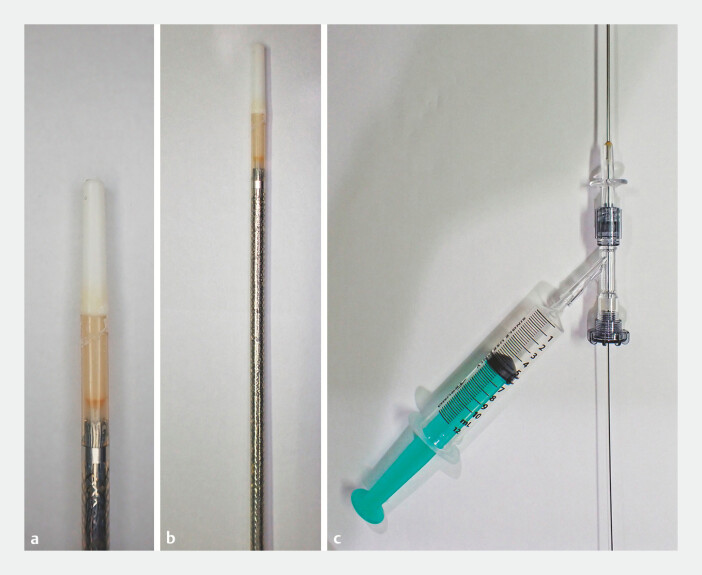
The catheter-like delivery system (BileRush Selective; Piolax Medical Devices) is preloaded with a 10 mm diameter uncovered metal stent and is compatible with guidewires 0.035 inches in size or smaller. This delivery system has a contrast injection function with a sufficient flow capability and has a diameter equivalent to small-caliber catheters (5.7 Fr). Additionally, it is equipped with a tapered tip that enhances penetration capability, similar to dilation catheters.

A 73-year-old man with MDBO and gastric outlet obstruction due to pancreatic cancer developed obstructive jaundice. The left intrahepatic bile duct was punctured transgastrically with a standard 19-G needle, and a 0.025-inch guidewire was advanced into the common bile duct (CBD). Subsequently, the catheter-like delivery system was inserted without prior fistula dilation, followed by cholangiographic evaluation with contrast injection. The guidewire was manipulated under the delivery system assistance to traverse the stricture and advance into the duodenum. The delivery system was then advanced over the guidewire, which enabled antegrade stent deployment across the stricture from the duodenum to CBD. Finally, a 7-Fr single-pigtail plastic stent was placed across the fistula. Fistula dilation was also unnecessary due to the bougie effect of the catheter-like delivery system ( Fig. 2 , Video 1 ). The patientʼs symptoms improved rapidly without adverse events.

**Fig. 2:**
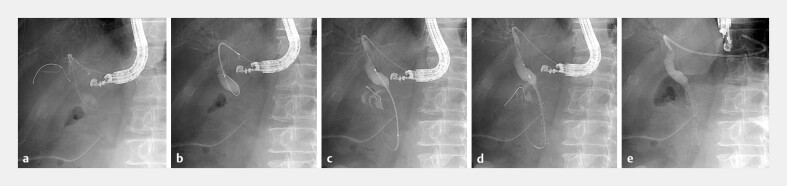
The left intrahepatic bile duct was punctured transgastrically with a standard 19-G needle (EZ shot 3 Plus; Olympus Medical Systems), and a 0.025-inch guidewire (VisiGlide2; Olympus Medical Systems) was advanced into the common bile duct ( **a** ). Subsequently, the catheter-like delivery system was inserted without prior fistula dilation, followed by cholangiographic evaluation with contrast injection ( **b** ). The guidewire was manipulated under the delivery system assistance to traverse the stricture and advance into the duodenum. The delivery system was then advanced over the guidewire ( **c** ), enabling antegrade stent deployment (10 × 80 mm) across the stricture from the duodenum to the common bile duct ( **d** ). Finally, a 7-Fr single-pigtail plastic stent was placed from the common hepatic duct to the stomach ( **e** ). Fistula dilation was also unnecessary due to the bougie effect of the catheter-like delivery system.

Endoscopic ultrasound-guided hepaticogastrostomy with antegrade stenting without fistula dilation step, using a catheter-like delivery system.Video 1

This technique represents a fully fistula dilation-free approach to EUS-guided transgastric drainage, which enhances safety by minimizing bile leakage and simplicity with reducing procedural time and step.

Endoscopy_UCTN_Code_TTT_1AS_2AD

## Notes

Endoscopy E-Videos https://eref.thieme.de/e-videos
*E-Videos* is an open access online section of the journal *Endoscopy* , reporting on interesting cases and new techniques in gastroenterological endoscopy. All papers include a high-quality video and are published with a Creative Commons CC-BY license. Endoscopy E-Videos qualify for HINARI discounts and waivers and eligibility is automatically checked during the submission process. We grant 100% waivers to articles whose corresponding authors are based in Group A countries and 50% waivers to those who are based in Group B countries as classified by Research4Life (see: https://www.research4life.org/access/eligibility/ ). This section has its own submission website at https://mc.manuscriptcentral.com/e-videos .
